# Deception and Cognitive Load: Expanding Our Horizon with a Working Memory Model

**DOI:** 10.3389/fpsyg.2016.00420

**Published:** 2016-04-07

**Authors:** Siegfried L. Sporer

**Affiliations:** Department of Psychology and Sports Science, University of GiessenGiessen, Germany

**Keywords:** deception detection, cognitive load, working memory model, schema theory, episodic memory

## Abstract

Recently, studies on deception and its detection have increased dramatically. Many of these studies rely on the “cognitive load approach” as the sole explanatory principle to understand deception. These studies have been exclusively on lies about negative actions (usually lies of suspects of [mock] crimes). Instead, we need to re-focus more generally on the cognitive processes involved in generating both lies and truths, not just on manipulations of cognitive load. Using Baddeley’s (2000, 2007, 2012) working memory model, which integrates verbal and visual processes in working memory with retrieval from long-term memory and control of action, not only verbal content cues but also nonverbal, paraverbal, and linguistic cues can be investigated within a single framework. The proposed model considers long-term semantic, episodic and autobiographical memory and their connections with working memory and action. It also incorporates ironic processes of mental control (Wegner, 1994, 2009), the role of scripts and schemata and retrieval cues and retrieval processes. Specific predictions of the model are outlined and support from selective studies is presented. The model is applicable to different types of reports, particularly about lies and truths about complex events, and to different modes of production (oral, hand-written, typed). Predictions regarding several moderator variables and methods to investigate them are proposed.

## Manipulating Cognitive Load: A Working Memory Perspective

Several studies have manipulated cognitive load under the assumption that this will increase differences between liars’ and truth-tellers’ behaviors (e.g., by reminding the sender to look into the eyes of the receiver). While cognitive load seems to have been successfully manipulated, and increased differences between liars and truth-tellers in a variety of behaviors have been found (see Vrij et al., 2010), to my knowledge these studies have not investigated the cognitive processes themselves that are induced by these manipulations (for the complexities involved in tapping these processes in the educational psychology/learning literature, see Beckmann, 2010). Manipulating cognitive load successfully is not the same as investigating the cognitive processes evoked by these manipulations. A more theoretical analysis is necessary to do so (Blandón-Gitlin et al., 2014).

A very simple manipulation to increase cognitive load is to have participants report about an event in reverse chronological order (Vrij et al., 2008). Note that this technique is also one of the components of the cognitive interview technique, which was included to enhance the number of details in recall of episodic events (Fisher and Geiselmann, 1992), not to add cognitive load. However in the context of the CL approach while lying is generally considered cognitively more taxing than truth-telling (e.g., Zuckerman et al., 1981), it is assumed that differences in observable behavior between liars and truth-tellers will be enlarged when participants tell an account in reverse order.

There is substantial evidence for this cognitive load assumption from polygraph as well as neuropsychological studies using rather simple (e.g., YES–NO) questions and response latencies as primary dependent measures (for critical reviews, see Verschuere et al., 2009; Gamer, 2011; Verschuere and De Houwer, 2011; Walczyk et al., 2013). Recently, Walczyk et al. (2014) have responded to some of these shortcomings by proposing an integrative cognitive model of deception for serious (high-stake) lies, which I return to below.

But what about other paraverbal and nonverbal cues, more complex questions and answers, and more complex verbal content cues as investigated with the Statement Validity Analysis and Criteria-based Content Analysis (Steller and Koehnken, 1989) and the reality monitoring approach (Sporer, 1997; Masip et al., 2005)?

**FIGURE 1:**
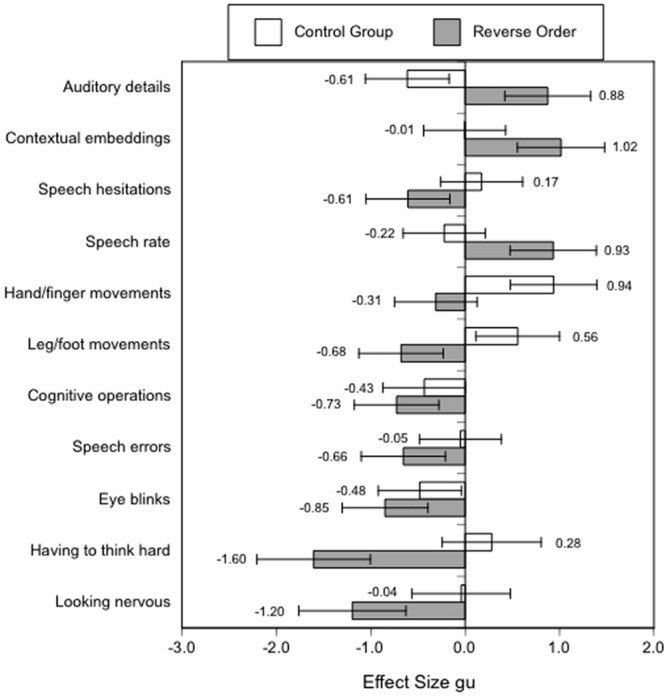
**Effect sizes gu [95% CIs] for nonverbal, paraverbal, and content cues to deception in the reverse order and control group of Vrij et al.’s (2008) study.** Positive values indicate an increase in truthful statements, negative values an increase in lies.

For example, in Vrij et al.’s (2008) study, 16 different, more complex measures were collected, but, unfortunately, data were only reported for nine measures, for which (marginally) significant effects for the expected Order by Veracity interaction were observed. I have calculated the effect sizes with the respective confidence intervals for the reported differences between lies and truths (see **Figure 1**).

Overall, the reported effects in Experiment 1 for the lie-truth differences in the reverse order condition are rather large in the predicted direction (more auditory details, contextual embeddings, and higher speech rates in true accounts, and more speech hesitations, cognitive operations, speech errors and eye blinks in lies. However, in line with the arousal approach of the four-factor theory (Zuckerman et al., 1981; Sporer and Schwandt, 2007) –but contrary to assumptions generally held by the cognitive load approach–, there were more leg/foot and non-significantly more hand/finger movements in lies than in truthful accounts in the reverse order condition. Furthermore, ratings by police officers in Experiment 2 indicated that liars appeared to have to think harder and to look more nervous than truth-tellers in the reverse order condition. It is clear that present accounts of “cognitive load theory” cannot serve as a single explanation for the reported findings. Rather, both the arousal approach and the attempted control approach appear necessary to account for the increase in nonverbal and paraverbal behaviors which are perceived generally as signs of “nervousness” (cf. Zuckerman et al., 1981; Sporer and Schwandt, 2006, 2007). There is also some recent research which shows that at least some nonverbal behaviors signaling nervousness may be indicative of deception in naturalistic settings such as crossing the borders at immigration (see Meservy et al., 2005).

Furthermore, in Vrij et al.’s (2008) control group, which presumably reflects constructing a lie without the added cognitive load, there was little support if any for the cognitive load approach. Hand/finger as well as leg/foot movements significantly increased, and there were fewer auditory details in truths compared to lies, with no significant differences for the remaining six dependent variables which were omitted from the report.

**FIGURE 2:**
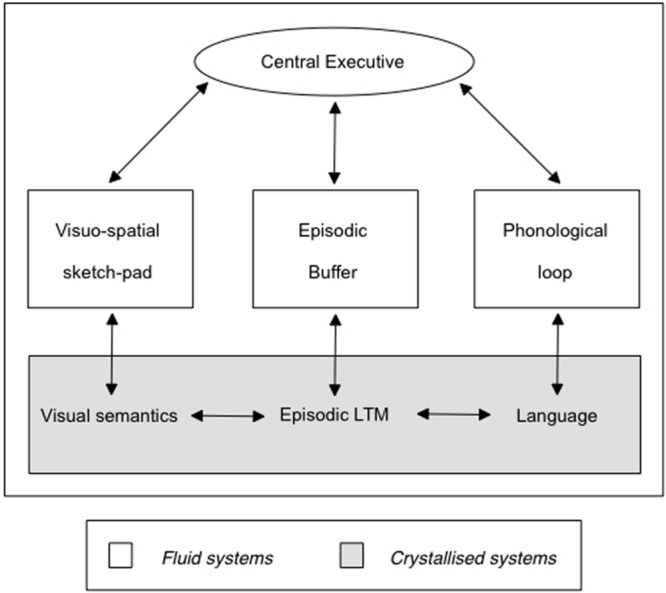
**Baddeley’s (2000) revised working memory model.** Note the interplay between episodic LTM and Episodic Buffer. The central executive is also considered important for the control of actions (Baddeley, 2000, 2012). Reprinted with kind permission from the author.

**FIGURE 3:**
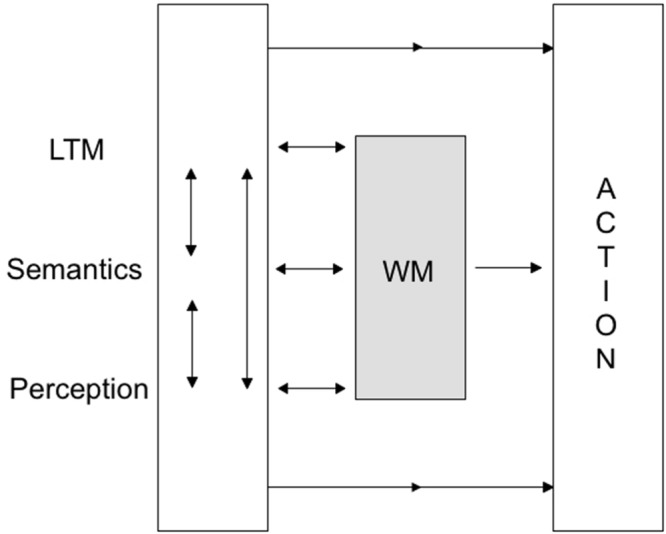
**Baddeley’s (2012) revised working memory model.** Note the direct paths from LTM, semantics and perception to action, in addition to the interactive pass via working memory. Reprinted with kind permission from the author.

However, in my view Vrij et al.’s (2008) findings could be reconciled with newer versions of Baddeley’s working memory model (in particular, Baddeley, 2000, 2007, 2012; see **Figures 2** and **3**). If we assume with Baddeley that the central executive in his working memory model is not only important for retrieval from long-term memory but also for the control of action (including movements of the extremities like hands, fingers, arms and legs), reducing cognitive resources by imposing dual task demands should make it more difficult for the sender to control signs of nervousness. These predictions would have to be tested with more stringent cognitive load manipulations as done in the dual task literature (e.g., responding to peripheral visual or auditory stimuli) besides the rather global reverse order instructions. Such cues could be manipulated either more mechanically (e.g., by random visual or auditory signals) or in an ecologically more valid fashion by (randomly determined) intermittent visual (e.g., shaking one’s head) or oral (e.g., a sigh) feedback signaling suspiciousness by an interviewer.

Furthermore, individual differences in working memory span should be considered to look for potential moderators in these effects (e.g., Engle and Kane, 2004). Finally, I expect the date of the experienced event to be reported upon as a potentially powerful moderator (cf. Sporer and Sharman, 2006) that has not been considered in recent laboratory studies where lying or telling the truth is usually about an event experienced immediately before. I expand on this aspect in the next section. Finally, investigators using the reverse order instruction should be aware that this may inadvertently introduce errors in reports (Dando et al., 2011). Any new method designed to improve detection of deception should always also be tested regarding negative side effects on accuracy, that is, whether or not it inadvertently increases errors of recall (see Sporer’s, 2008, integrative model of eyewitness testimony).

## Schemata as a Basis for the Construction of Lies and Truths

Both in the basic memory literature on prose recall, and more pertinently in the eyewitness literature, it has been amply documented that people do not recall episodes verbatim but merely the “gist” (e.g., of a conversation). One of the reasons why I find Baddeley’s working memory model particularly attractive for the understanding of lies and truths is that it also incorporates links between long-term (both semantic and episodic) memory and the active working memory components (phonological loop, visuo-spatial sketchpad and episodic buffer). What knowledge do liars draw upon in constructing their lies? Obviously, if one has not had a personal experience of an event or a sequence of action one has to draw upon general declarative and procedural knowledge to construe this event (cf. Herrmann’s, 1982, model of speech production). If people have not had related or similar particular experiences they have stored in their episodic memory and which they can substitute for the real event they are supposed to talk about they have to rely upon their semantic memory to construe such an episode. How is this semantic memory organized, and what retrieval strategies do people use in such a case?

While there may be many answers to this question, associative network models (e.g., Anderson, 1983; Smith, 1998) and fuzzy trace theory (e.g., Reyna and Brainerd, 1995) seem to be particularly relevant. Although these models are more descriptive, they have proven useful in studying eyewitness testimony (e.g., Tuckey and Brewer, 2003b; Kleider et al., 2008), and certain predictions outlined below are in line with these models.

One approach that has been quite popular in the (social) cognition literature has described this knowledge in terms of scripts (Schank and Abelson, 1977) or schemata (e.g., Brewer and Nakamura, 1984; for reviews, see Fiske and Taylor, 1991; Rojahn and Pettigrew, 1992; Stangor and McMillan, 1992). This approach has been applied to the assessment of credibility of statements by Köhnken (1990) and Sporer and Küpper (1995).

It is assumed that a schema or an action script summarizes the characteristic components of a sequence of actions such as ordering food in a restaurant, or a bank robbery. What is stored are not the individual components (e.g., reading the menu, the waitress taking notes...) but only a “pointer” to the schema (Graesser, 1981). Supposedly, this serves as a quick and economic way to store and retrieve schema-consistent information. With repeated exposure to script-like action sequences, schema-consistent information becomes more generic, abstracting from specific episodes. With increasing retention interval, schema-irrelevant details of concrete episodes fade from memory and are less likely recalled (Tuckey and Brewer, 2003a,b; see the meta-analyses by Rojahn and Pettigrew, 1992, and Stangor and McMillan, 1992).

On the other hand, schema-inconsistent information, that is, deviations from the schema that may be unique to a specific episode is more likely to draw attention at encoding (and may also be rehearsed more frequently, e.g., when talking to somebody about the incident; cf. the literature on autobiographical memory on the role of rehearsal of significant life events: e.g., Conway, 1990; Thompson et al., 1996), and hence is postulated to create a stronger memory trace. According to fuzzy trace theory, both schema-consistent and schema-inconsistent information are stored as gist traces, while schema-irrelevant information is stored at a verbatim level, which is more likely to be subject to decay (Reyna and Brainerd, 1995; Tuckey and Brewer, 2003b). For example, the gist or meaning of a conversation will be likely correctly recalled whereas memory for specific expressions as part of an utterance will not (for reviews, see Davis and Friedman, 2007; Read and Connolly, 2007).

A direct implication for the construction of lies is that lies will be composed of typical or schema-consistent information generally available to the story-teller. To the extent that true experiences are unique and distinctive, that is, contain schema-inconsistent information, this information will be more likely recalled, while schema-irrelevant information is less likely to be available over time (see also the von Restorff effect: von Restorff, 1933). A related distinction between central and peripheral details of an event has also proven useful in the eyewitness memory literature (Ibabe and Sporer, 2004).

In support of these assumptions, invented accounts in real world cases have been characterized by their “abstractness” (Bender, 1987) or as “schematic” (Arntzen, 1993). Hence, invented accounts are likely to lack the richness of details characteristic of true stories (Undeutsch, 1967; Steller and Koehnken, 1989; Arntzen, 1993; Sporer, 1997, 2004). Although the lack of details cannot be coded directly in an account, and hence cannot serve as a lie criterion, ratings of perceived “abstractness” are possible and can be considered on a continuum from abstractness to quantity/quality/richness of details. Using Semin and Fiedler’s (1991) linguistic category model, we could show that invented accounts of personally significant life events were rated as much more abstract than those of self-experienced events (*d* = 0.48; Sporer et al., 2003).

In contrast, schema-inconsistent or atypical information should be particularly diagnostic for truth-based accounts. Atypical information has to be stored separately and is likely to be even better recalled than typical or schema-irrelevant information (Graesser and Nakamura, 1982; Tuckey and Brewer, 2003a,b).

With respect to credibility criteria, truth-based accounts have been characterized by superfluous and unusual details, unexpected negative complications and so forth (Arntzen, 1993; Steller and Koehnken, 1989; Sporer, 1997, 2004). However, with increasing delay (retention interval) schema-irrelevant details are also likely to be forgotten more readily (e.g., Tuckey and Brewer, 2003a,b). Graesser and Nakamura (1982) postulate that the benefit of atypical information will decay with increasing retention interval. A direct corollary for the construction of lies would be that with increasing temporal distance between the (supposed) perception of an event and the recall attempt differences between truth-based and fictitious accounts are likely to fade (Köhnken, 1990). An exception may be true memories containing highly salient, “unusual” details which may be more frequently rehearsed (and retold) and hence better remembered. By comparison, memories of lies that do not contain these types of elements will be less likely rehearsed. These contrary predictions ought to be examined in future research.

To summarize, the schema theoretical approach presents an interesting framework that may help us to understand some aspects of the construction of complex lies. To the extent that events are not self-experienced story-tellers need to rely upon general semantic as well as schematic knowledge to present sequences of events. The older the memory of an event, the more difficult retrieval will be, perhaps levelling differences in content cues between lies and truths that may still be visible with younger memories (Sporer and Sharman, 2006). Also, retrieval of older episodic memory traces may rely more on schemata and scripts, thus furthermore reducing differences between lies and truths.

The working memory model advocated here should also be applicable to a broad range of lies and truths. It should be tested with different types of lies (not just denials of negative actions as in staged crime paradigms, partial concealment and omissions) but also lying about positive events (e.g., past accomplishments), (false) alibis and (false) confessions, or alleged traumatic events (of physical or sexual abuse, or torture by asylum seekers).

It is not clear whether this model will be applicable to lying about intentions where a reverse order instruction did not lead to an increase in detection accuracy but merely to a more lenient response bias (Fenn et al., 2015). The model may also be less applicable to less serious lies in everyday life (DePaulo et al., 1996) where speakers may simply adjust their utterances “on the fly” (cf. McCornack et al., 2014, IMT2). But even when speech utterances are produced at high speed, editing out information not to be revealed does involve both the phonological loop and the central executive, which may lead to slips and slower response latencies (cf. Gathercole and Baddeley, 1993, model of working memory and speech production and the discussion of slips below).

## Mode of Production

A very simple question that could be quite crucial not only in detection of deception research but more generally in the eyewitness literature is the mode of production in which a sender communicates his or her message. While communication researchers have realized for a long time that the medium with which a message is conveyed (oral, hand-written, typed) may affect cues to deception, this issue has been largely ignored in recent studies. For example, one of the major differences between studies analyzing content cues using CBCA and RM approaches on the one hand and SCAN on the other hand is that the latter rely on hand-written statements by communicators.

There is some (although somewhat equivocal) evidence from basic experimental memory, eyewitness memory, and neuropsychological studies that recall of text and/or episodic memories may depend on the mode in which statements are delivered (Horowitz and Newman, 1964; Moscovitch, 1994; Rosenthal, 2002; Kellogg, 2007). Relatedly, research on self-reported medical histories has shown that the information provided may differ whether the information is provided in a self-administered questionnaire or in an in-person interview (Bergmann et al., 2004). In eyewitness research, Sauerland and Sporer (2011) have recently shown that the superiority of oral vs. written reports may only hold for central details (e.g., facial descriptions of perpetrators and central aspects of crime descriptions) both with respect to quantity and accuracy but not for peripheral details (e.g., clothing) where the effect may even be reversed.

Presumably, writing places higher demands on working memory than speaking because writing is slower, less practiced, and entails the activation of graphemic representations for spelling words (Kellogg, 2007).

When cognitive load is high, performance for effortful retrieval from long-term memory is reduced (Moscovitch, 1994). Consequently, memory retrieval should benefit from a reduction in cognitive load. This assumption has recently found ample support from studies by Vredeveldt et al. (2011) in which interviewed eyewitnesses were asked to close their eyes at the time of retrieval. When engaged in a cognitively more demanding task (written description) witnesses may thus choose to describe primarily those details that require less effort (clothing) rather than those that require more effort (face). If, however, cognitive load is lower (spoken descriptions) witnesses may have more working memory capacity available to describe those details that require more effort. Applying these ideas to deception research, it appears necessary to be more specific when formulating research hypotheses, taking the distinction between central and peripheral details as a moderator into account (for a general discussion of central vs. peripheral details, see Ibabe and Sporer, 2004).

**FIGURE 4:**
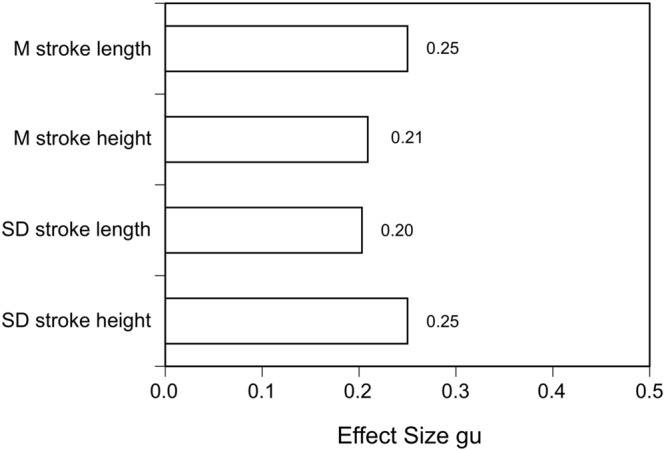
**Effect sizes gu for means and standard deviations of stroke length and width (data from Luria and Rosenblum, 2010)**.

On the other hand, writing a report of an event by hand or typing it without time pressure may allow the writer to take their time to prepare an outline first, deliberate each sentence and its possible consequences (for example, when filling out an insurance claim form). Nonetheless, in an intriguing study Luria and Rosenblum (2010) found differences between lies and truths in hand-written reports (in Hebrew script). Lies showed longer and more variable mean stroke lengths and longer and more variable mean stroke heights than true accounts (albeit with rather small effect sizes; see **Figure 4**). Of course, this study needs to be replicated with Roman hand-writing in different Western countries. In another study with a large number of accounts of self-experienced vs. invented personally significant events written down in hand-writing immediately or one week after being given the instructions showed that significant differences in reality monitoring criteria could be detected (Sporer and Küpper, 1995).

As more and more communication takes place via computers or smart phones, future research should also consider this medium for analyzing linguistic and content cues to deception (cf. Hauch et al., 2015). From a cognitive load perspective, we would expect more writing errors when typing a lie than when composing a truthful message (a hypothesis not supported in Hauch et al.’s, 2015, meta-analysis). On the other hand, based on DePaulo’s self-presentational perspective (DePaulo et al., 2003), liars might be more self-aware and deliberate than truth-tellers; hence, they may edit their typing errors more carefully. More direct evidence comes from a study by Derrick et al. (2012) that showed liars were significantly more likely to edit their words on the keyboard (e.g., in using the backspace and delete button) than truth-tellers (*d* = –0.12 [–0.19; –0.05]). Whether or not their edits were aimed at correcting explicit typing errors or not, was not investigated and should be investigated more closely in future studies. However, when typing a message (as in online chats or emails), differences in typing skill may be an important moderator variable resulting in various levels of cognitive load.

## Social, Contextual, Cultural, and Personality Factors

Besides cognitive factors, social and contextual factors may also affect the number and quality of responses (Rosenthal, 2002; Bergmann et al., 2004). Specifically, interviewers in the spoken conditions may have subtly (although instructed not to) signaled to the participants to speak more about central details than about peripheral ones. It is known that such influences occur (Rosenthal, 2002) and that people are not aware of exerting them (Greathouse and Kovera, 2009). This could be tested by interviewing some participants in person (and videotaping them) while other participants make their spoken statements with the aid of a voice recorder, that is, with no interviewer present. If the spoken advantage is primarily due to social factors, their influence should be diminished in the voice recorder only condition. As the process of videotaping itself may increase cognitive load (participants are often quite apprehensive about being videotaped; cf. the literature on objective self-awareness: Wicklund, 1975), comparisons with hidden cameras may also be necessary. Within this paradigm, the role of cognitive factors, on the other hand, could be tested by manipulating cognitive load in a dual task manner during spoken reports. Cross-cultural factors, for example when answering questions in a foreign language (with or without an interpreter) in interviews of terrorism suspects or asylum seekers will also have to be taken into account as “natural variations” in cognitive load as a function of language ability (Evans et al., 2013).

Differences in verbal intelligence and linguistic abilities may also moderate the ability of speech (or written) production and thus indirectly affect the relationship between cognitive load and lie production. There is evidence from autobiographical memory research that the origins of events retrieved by bilinguals varied as a function of the interview language (Marian and Neisser, 2000).

## Lies and Truths About Wrongful Deeds Vs. Positive Events and Actions

Probably determined by funding bodies, recent research has exclusively focused on lies by suspects (or witnesses) about criminal behavior or intentions. Most of the events to be reported on are also negative in emotional tone. However, an encompassing theory of lie and truth production must not only focus on such negative actions and events but should also be able to explain reports of positive events and actions (e.g., a job applicant emphasizing his or her accomplishments in the past at a particular task). In particular, theorizing about the role of inhibition in presenting a lie has explicitly postulated that the respondent has knowledge about his or her true actions which need to be “suppressed”, thus requiring mental resources (Walczyk et al., 2003, 2005; Blandón-Gitlin et al., 2014; for reviews, see Walczyk et al., 2013). Rather than focusing on suspicious nonverbal signs, investigators ought to pay more attention to what a person is saying, that is, the content of a message (Sporer, 2004; Reinhard et al., 2011; Ormerod and Dando, 2014).

## Comparing the Present Model with Other Models

Recently, several models of lie production have been proposed (Walczyk et al., 2005, 2013; Levine, 2014; Levine and McCornack, 2014; McCornack et al., 2014; Walczyk, 2014). These models vary widely in scope and the type of deception phenomena they could potentially explain.

In his truth default theory (TDT), Levine (2014) made the important point that in most day to day interactions, communicators will tell the truth, thus making it particularly difficult to detect the few deceptive messages that ever occur (or deceptive parts of a message), given their low base rates.^1^ For specific types of messages, in particular, lies about crimes (usually in the form of false denials or false alibis) the base rate may be higher when the expected consequences of truth-telling are high (serious lies). Nonetheless, in line with McCornack’s Information Manipulation Theory 2 (IMT2), it is probably safe to assume that for most lies the easiest way to proceed, requiring the least cognitive effort, is to stay as close as possible to the actual truth. This may be done consciously by planning, designing and rehearsing a deceptive message, which together are often described as deception strategies (cf. Leins et al., 2013; Masip and Herrero, 2013). In one of our studies on a complex, personally significant life event (participants’ driving exam) a large proportion of participants admitted in a postexperimental questionnaire that they had used episodic memories from their own driving lessons to construct their accounts, despite the clear instruction to “freely invent” their story (Sporer and Walther, 2006). Even if participants did not intentionally choose specific elements from driving lessons or scripts for a driving exam derived from conversations with others, the frequency of recent driving experiences preparing for the exam would create many recently activated associative links which would now be readily available for incorporating them.

But such “content borrowing” may also happen unconsciously, without any planning or strategic intention (see also McCornack et al., 2014, IMT2). As Lampinen et al. (2005) have shown with simple word list paradigms, people may create vivid false memories that are difficult to discern from true memories. While the basic experimental paradigms used in this and similar studies are a far cry from the complexity of narrating autobiographical life events, numerous studies demonstrating false memories as a consequence of suggestive questioning or other misleading postevent information make it clear that complex memories may be constructed without a veridical basis (e.g., Read, 1996; Loftus, 2005; Lindsay, 2008).

But content for a convincing story is not just borrowed. Any story to be told, whether truthful or invented, must draw on long-term memory. This way, the present model is not a mere working memory model. Reconstructive memory processes are at work both when people recreate a true experience from episodic and semantic memory and when they try to create (“invent”) a story of an event they have never experienced at all, or not in the way presented.

Specific cues activated by the retrieval context or the questions asked by the recipient or an interviewer serve to engage retrieval processes which activate relevant elements from long-term memory and make them available in working memory. In doing so, schemata and scripts from similar or related experiences may also be engaged to search in long-term memory to speed retrieval processes and to fill gaps when certain details are not readily available.

In working memory, focusing attention on the retrieved contents allows to engage in control processes that determine which information is to be transformed into verbal utterances. Information is to be presented or withheld in line with Gricean rules of conversation (quantity, quality, relation, manner; Grice, 1975; see also Fiedler, 1989a,b; McCornack et al., 2014). Note that Gricean rules play a role both in truthful and deceptive communication but different principles are violated to different degrees in specific types of lies (see McCornack et al., 2014, for a detailed discussion).

### Differences Between the Models

My (Working-)Memory Model clearly goes beyond Walczyk et al.’s (2003) ADCM and Walczyk et al.’s (2005) Tri-Con models by focusing more on complex lies. Thus, it is closer to, with many points of overlap, with Walczyk et al.’s (2009) ADCM-Revised and his most recent Activation-Decision-Construction-Action Theory (ATCAT; Walczyk et al., 2014). Walczyk et al. (2014) try to overcome many of these shortcomings in their earlier models by incorporating some of the propositions of McCornack et al.’s (2014) IMT2 and Sporer and Schwandt’s (2006, 2007) earlier working memory model.

Note, however, that McCornack’s IMT2 and Walczyk’s ATCAT models really focus on different types of lies: While McCornack focuses on relatively frequent, everyday lies that are likely to be created and edited “on the go” during the process of speech production, Walczyk and myself have more serious lies in mind. Thus, the McCornack and Walczyk two models complement each other, like two ships passing each other in the night. One of the major differences of my own and these models is that my model is also supposed to be applicable to lies about positive events not requiring any suppression of the truth. Furthermore, my model is also supposed to be applicable to lies produced not only vocally but also in writing (hand-writing or typed). Furthermore, I assume that strategic aspects of both truth-tellers and liars affect both verbal content and nonverbal and paraverbal behaviors. Serious lies are often planned, prepared and rehearsed, and both truth-tellers and liars engage self-presentational strategies to appear credible (what Köhnken, 1990, refers to secondary deception). In contrast, in McCornack’s IMT2 the role of planning and strategic behavior takes only a backseat. Finally, my model is informed by working memory and retrieval processes in recalling self-experienced events and how they are reported. Thus, the focus is more on truth-telling than on the production of lies.

### Predictions and Empirical Support for My Model

One of the predictions from the present (working-)memory model that is in line with all these models is that liars will take longer to respond when answering questions. This proposition has primarily been investigated in polygraph type studies (see DePaulo et al., 2003; National Research Council, 2003), and more recently in studies on the Control Question Technique (CGT) and the Concealed Information Test (CIT; formerly labeled “Guilty Knowledge Test”; for critical reviews, see Verschuere and De Houwer, 2011; Walczyk et al., 2013). Most of these studies, however, rely on simple Yes-No questions, perhaps also on simple, one or two word utterances, that do not reflect the type of complex lies I am addressing in my model. These models also implicitly assume that something negative (a mock crime) is to be hidden, using some form of inhibition processes (Gombos, 2006).

I fully agree with Walczyk (2014) that a comprehensive model of lie production ought to incorporate a theory of mind concept as postulated long ago by researchers on children’s deception (Wimmer and Perner, 1983; see the meta-analysis by Wellman et al., 2001). The very definition of a lie as intending to induce a belief (subjectively believed to be false) in another person presumes the attribution of the recipient’s mental state as well as the intention to change that belief. Where I disagree, however, is the one-sided discussion on lie production that prevails in practically all contemporary accounts. When researchers investigate differences between lies and truths these differences may come about (1) by lie-specific cognitive (and social) mechanisms that most researchers have investigated. However, these differences may also be a function of (2) the better accessibility of episodic (and semantic) memory traces truth-tellers can take advantage of. My own model focuses more on verbal content characteristics that become apparent in free recall and open ended questions which initiate the retrieval processes in truth-tellers. Associative network models (Anderson, 1983; Smith, 1998) provide the basis for my assumptions which are supported both by basic memory research that encompasses research on working memory processes with those involved in episodic memory, semantic and autobiographical memory (see **Figures 2** and **3**; Conway, 1990; Conway and Pleydell-Pearce, 2000; Baddeley, 2012; Baddeley et al., 2015). The frequency of access to traces of experienced events as well as the number of (self-generated) cues to one’s episodic, in particular significant autobiographical, memories and the accompanying rehearsal processes (cf. Conway, 1990; Sporer, 2004) strengthen these traces and speed up their access.

**FIGURE 5:**
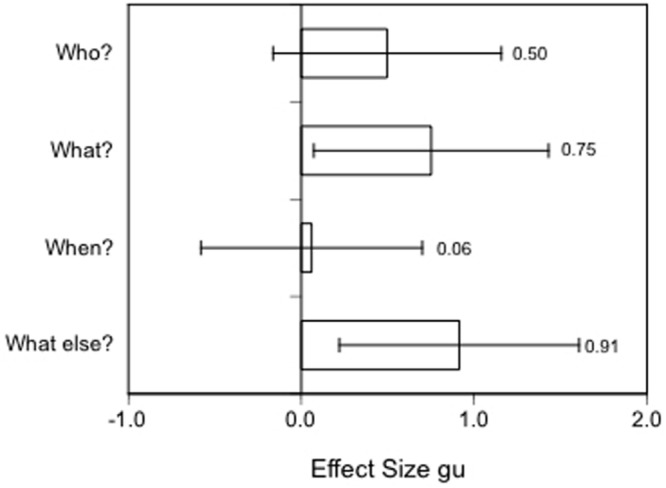
**Effect sizes gu [95% CIs] for response latencies to followup questions in the interview.** Positive values indicate longer response latencies in lies (data from Sporer and Petermann, 2011).

As an example, in a quasi-experimental study by Sporer and Walther (2006) participants first reported freely about a driving exam they had either taken, or were about to take in the near future; half of the participants were also asked specific, open-ended follow up W-questions on Who, What, When, and What else (Camparo et al., 2001). In a re-analysis of Sporer and Walther’s audiotapes by Sporer and Petermann (2011), response latencies were determined from voice-spectrum analyses by calculating the times between the end of a question and the beginning of the corresponding answers. Although the sample size for these comparisons were small, results indicated medium to large effect sizes for response latencies as a function of truth status. Liars took substantially longer to answer three of these four relatively open W-questions demonstrating than truth-tellers (**Figure 5**).

Note that in this study there was no “truth to be suppressed” as the event to be described was still in the future. Also, all participants were given the opportunity to prepare their story beforehand (which, on average, took 2.25 min, *SD* = 0.85). An implication of this finding is that “unexpected questions” or questions within the “strategic use of evidence” technique should make these differences even larger but not because retrieval of “false” memories is slowed down but more likely because retrieval of elements based on self-experience will be speeded up.

### Ironic Effects of Blocking of Unwanted Thoughts and Lies of Omission

The fruitfulness of associative network models becomes also evident when we consider lies of omission, that is, (intentionally or unconsciously) leaving out information from an account (see McCornack’s IMT2). According to the prevailing models discussed above, particularly when the lie is about a wrong-doing or an attitude opposite to the one held by the storyteller, and/or when the interviewee is a guilty criminal suspect, the storyteller must also suppress thoughts about the truth (Gombos, 2006). This continued suppression of unwanted thoughts may inadvertently preoccupy his or her thinking (Pennebaker and Chew, 1985; see also Lane and Wegner, 1995, model of secrecy).

While McCornack et al. (2014) postulate that this occurs rather spontaneously during message production, Wegner’s (1994) ironic processing theory suggests that the very process of suppressing a thought actually strengthens it. Note that this proposition is also in full agreement with my assumption that the number and frequency of activated paths in an associated network (Anderson, 1993; Smith, 1998) makes them more accessible and ready for output.

Wegner (1994) assumes two types of processes: an intentional operating process and an ironic monitoring process. Based on early theorizing in cybernetics, goal-directed behavior results from a cycle of “operate” and “test” mechanisms in a test-operate-test-exit (TOTE) unit (Miller et al., 1960; see also Köhnken’s, 1990, application of these mechanisms to deception). According to Wegner, the operating process “orients the person toward items of sensation and memory that are consistent with the desired state of mind” (p. 37). This process is effortful and consumes resources and hence is subject to interferences from other attentional demands. In contrast, the monitoring process is not conscious, requires little mental effort and searches continuously for sensations and thoughts that are consistent with the achievement of mental control. The theory has received empirical support with various paradigms, from stereotype suppression, self-presentation, and deception.

Applied to deception, a communicator attempts to control his or her thoughts and behaviors in order to appear credible. In the background, the ironic monitoring continuously searches for thoughts and behaviors that might counteract this intention. As the operating process requires more effort than the monitoring process, situational factors like time pressure and cognitive demands but also dispositional factors like low working memory capacity will impair the operating process more than the ironic processes.

As the research following Wegner’s theory shows, repeatedly thinking that we should or must not think certain thoughts, utter them in speech, or show them in overt action actually increases the frequency of these behaviors. Importantly for the present discussion, these effects are stronger in dual task paradigms where cognitive resources are depleted by concurrent tasks (e.g., when trying to hold a pendulum steady and simultaneously counting backwards by threes from 1000; Wegner et al., 1998; see also Wegner, 2009). In the domain of deception among partners, survey data provided correlational evidence that past “hot crushes” (desired romantic relationships that never materialized and were most often thought about) preoccupied them more and were more secret than relationships less often thought about (Wegner et al., 1994). In an ingenious follow-up laboratory experiment in which one team (a dyad of participant strangers) was encouraged to touch their opposite sitting team partner either secretly or with knowledge of the opposing team (another dyad), preoccupation of thought and subsequent attractiveness ratings were highest for the secret contact person compared to the other conditions. Other studies have also applied Wegner’s model to cognitive consequences of keeping secrets (e.g., Lane and Wegner, 1995; Lane and Liersch, 2012; for a comprehensive treatment of the relationship between keeping secrets and speech production, see Spitznagel, 1998). Furthermore, Wardlow Lane et al. (2006) linked this model to the theory of mind concept, investigating in detail the failure of speakers’ control over leaking private information during language production. Wegner’s theory as well as the studies in support of it, indicate that it is more difficult to intentionally omit or suppress information the recipient should not know about. Although some of the paradigms used are rather simple, I would like to see further tests with more complex contents and with different types of topics.

Compatible with Wegner’s model are also findings from an ingenious research program by Bredenkamp and colleagues who investigated slips of the tongue (including “Freudian” slips, Freud, 1901/1954) and slips of action (see Baars, 1992) in a series of experiments (for a summary, see Hamm and Bredenkamp, 2004). The paradigms used were rather simple, examining spoonerisms (transpositions of [usually initial] sounds of two or more words) with word pairs and should not be seen as conclusive demonstrations of “Freudian” slips (see Baars et al., 1992). Nonetheless, the theoretical rationale by Hamm and Bredenkamp (2004) combines Baddeley’s (2000) working memory model with Gathercole and Baddeley’s (1993) model of speech production. The elegance of Hamm and Bredenkamp’s experiments lies in the fact that they involve direct independent manipulations of the phonological loop and the central executive that affect both the production of spoonerisms and response latencies. Differences in response latencies also play a central role in Walczyk and colleagues’ theorizing as well as in the newer “memory detection” literature (Walczyk et al., 2009, 2013; Verschuere and De Houwer, 2011).

## Conclusion

My major concern with recent studies on cognitive load is that despite the creativity of many of the interventions suggested these are not derived from general theoretical principles. Also, many of the dependent variables measured are study-specific (e.g., sometimes general details, sometimes more specific details regarding spatial arrangements, “inconsistencies”, or even paraverbal or nonverbal behaviors and impressions). We need a theory of cognitive load that specifies both the cognitive processes induced by these manipulations as well as a taxonomy of outcomes (cues) likely to be affected by them. Also, the other components of the four-factor model still need to be considered.

Furthermore, note that many of the theoretical arguments I drew upon to suggest changes in paradigms were not derived from theoretical analyses of the cognitive processes involved in constructing lies but from basic and applied memory research which is usually concerned with studying “truthful” (but perhaps error-prone) recall from episodic and/or autobiographical memories. While working memory may be particularly taxed in constructing lies, we need to compare the processes involved in generating both truthful and invented accounts that involve the interplay of attention, working memory, and long-term semantic and episodic memory as well as the production of speech and action.

## Author Contributions

The author confirms being the sole contributor of this work and approved it for publication.

## Conflict of Interest Statement

The author declares that the research was conducted in the absence of any commercial or financial relationships that could be construed as a potential conflict of interest.

## Footnotes

1 An implication of Levine’s TDT is that recipients will show a truth bias when evaluating communicators’ messages. Street (2015) has recently challenged Levine’s TDT assumption, arguing that the truth bias may not be a distorting “bias” but rather an experience-based heuristic that is likely to work well in many everyday life contexts. When context-general information suggests that in this type of context people will likely tell the truth it may well be adaptive to assume that people will tell the truth under these circumstances. To the extent that this is the case, detection accuracy will be higher than previously assumed. However, in other contexts, base rates of lying may be higher. Consequently, it would be adaptive to adopt a lie bias under these circumstances. Because both TDT and Street’s (2015) model focus more on the detection of deception than on lie production discussed here, I will not expand on this point.

## References

[B1] Anderson J. R. (1983). A spreading activation theory of memory. *J. Verb. Learn. Verb. Behav.* 22 261–295. 10.1016/S0022-5371(83)90201-3

[B2] Anderson J. R. (1993). Problem solving and learning. *Am. Psychol.* 48 35–44. 10.1037/0003-066X.48.1.35

[B3] Arntzen F. (1993). *Psychologie der Zeugenaussage [Psychology of Testimony].* Munich: Beck.

[B4] Baars B. J. (ed.) (1992). *Experimental Slips, and Human Error. Exploring the Architecture of Volition.* New York, NY: Plenum Press.

[B5] Baars B. J. Cohen J. Bower G. H. Berry J. W. (1992). “Some caveats on testing the Freudian slip hypothesis: problems in systematic replication,” in *Experimental Slips and Human Error* ed. Baars B. J. (New York, NY: Plenum Press) 289–313.

[B6] Baddeley A. Eysenck M. W. Anderson M. C. (2015). *Memory.* London: Psychology Press.

[B7] Baddeley A. D. (2000). The episodic buffer: a new component of working memory? *Trends Cogn. Sci.* 4 417–423. 10.1016/S1364-6613(00)01538-2 11058819

[B8] Baddeley A. D. (2007). *Working Memory, Thought and Action.* Oxford: Oxford University Press.

[B9] Baddeley A. D. (2012). Working memory: theories, models, and controversies. *Annu. Rev. Psychol.* 63 1–29. 10.1146/annurev-psych-120710-100422 21961947

[B10] Beckmann J. F. (2010). Taming a beast of burden–On some issues with the conceptualisation and operationalisation of cognitive load. *Learn. Instruct.* 20 250–264. 10.1016/j.learninstruc.2009.02.024

[B11] Bender H.-U. (1987). *Merkmalskombinationen in Aussagen [Criteria Combinations in Eyewitness Statements].* Tübingen: J. C. B. Mohr.

[B12] Bergmann M. M. Jacobs E. J. Hoffmann K. Boeing H. (2004). Agreement of self-reported medical history: comparison of an in-person interview with a self-administered questionnaire. *Eur. J. Epidemiol.* 19 411–416. 10.1023/B:EJEP.0000027350.85974.47 15233312

[B13] Blandón-Gitlin I. Fenn E. Masip J. Yoo A. H. (2014). Cognitive-load approaches to detect deception: searching for cognitive mechanisms. *Trends Cogn. Sci.* 18 441–444. 10.1016/j.tics.2014.05.004 25168448 PMC4309739

[B14] Brewer N. Nakamura G. V. (1984). “The nature and functions of schemas,” in *Handbook of Social Cognition* Vol. 1 eds Wyer R. S. Srull T. K. (Hilldale, NJ: Lawrence Erlbaum) 119–160.

[B15] Camparo L. B. Wagner J. T. Saywitz K. J. (2001). Interviewing children about real and fictitious events: revisiting the narrative elaboration procedure. *Law Hum. Behav.* 25 63–80. 10.1023/A:1005691926064 11276862

[B16] Conway M. A. (1990). *Autobiographical Memory.* Milton Keynes: Open University Press.

[B17] Conway M. A. Pleydell-Pearce C. W. (2000). The construction of autobiographical memories in the self-memory system. *Psychol. Rev.* 107 261–288. 10.1037/0033-295X.107.2.261 10789197

[B18] Dando C. J. Ormerod T. C. Wilcock R. Milne R. (2011). When help becomes hindrance: unexpected errors of omission and commission in eyewitness memory resulting from change temporal order at retrieval? *Cognition* 121 416–421. 10.1016/j.cognition.2011.06.015 21861997

[B19] Davis D. Friedman R. D. (2007). “Memory for conversation: the orphan child of witness memory researchers,” in *Handbook of Eyewitness Psychology: Memory for Events* Vol. 1 eds Toglia M. P. Read J. D. Ross D. F. Lindsay R. C. L. (New York, NY: Lawrence Erlbaum Associates) 3–52.

[B20] DePaulo B. M. Kashy D. A. Kirkendol S. E. Wyer M. M. Epstein J. A. (1996). Lying in everyday life. *J. Pers. Soc. Psychol.* 70 979–995. 10.1037/0022-3514.70.5.979 8656340

[B21] DePaulo B. M. Lindsay J. J. Malone B. E. Muhlenbruck L. Charlton K. Cooper H. (2003). Cues to deception. *Psychol. Bull.* 129 74–118. 10.1037/0033-2909.129.1.74 12555795

[B22] Derrick D. Meservy T. Burgoon J. Nunamaker J. (2012). “An experimental agent for detecting deceit in chat-based communication,” in *Proceedings of the Rapid Screening Technologies, Deception Detection and Credibility Assessment Symposium* Grand Wilea.

[B23] Engle R. W. Kane M. J. (2004). “Executive attention, working memory capacity, and a two-factor theory of cognitive control,” in *The Psychology of Learning and Motivation* ed. Ross B. H. (San Diego, CA: Elsevier Academic Press) 145–199.

[B24] Evans J. R. Michael S. W. Meissner C. A. Brandon S. E. (2013). Validating a new assessment method for deception detection: introducing a psychologically based credibility assessment tool. *J. Appl. Res. Memo. Cogn.* 2 33–41. 10.1016/j.jarmac.2013.02.002

[B25] Fenn E. McGuire M. Langben S. Blandón-Gitlin I. (2015). A reverse order interview does not aid deception detection regarding intentions. *Front. Psychol.* 6:1298 10.3389/fpsyg.2015.01298 PMC4553365 26379610

[B26] Fiedler K. (1989a). Lügendetektion aus alltagspsychologischer Sicht [Lie detection from a commonsense point of view]. *Psychol. Rundsch.* 40 127–140.

[B27] Fiedler K. (1989b). “Suggestion and credibility: lie detection based on content-related cues,” in *Suggestion and Suggestibility* eds Gheorghiu V. A. Netter P. Eysenck H. J. Rosenthal R. (Berlin: Springer-Verlag) 323–335.

[B28] Fisher R. P. Geiselmann R. E. (1992). *Memory-Enhancing Techniques for Investigative Interviewing: The Cognitive Interview.* Springfield: Charles C. Thomas.

[B29] Fiske S. T. Taylor S. E. (1991). *Social Cognition.* New York, NY: McGraw-Hill.

[B30] Freud S. (1901/1954). *Zur Psychopathologie des Alltagslebens [The Psychopathology of Everyday Life].* Frankfurt am Main: Fischer Taschenbuch Verlag.

[B31] Gamer M. (2011). “Detecting of deception and concealed information using neuroimaging techniques,” in *Memory Detection* eds Verschuere B. Ben-Shakhar G. Meijer E. (Cambridge: Cambridge University Press) 90–113.

[B32] Gathercole S. Baddeley A. (1993). *Working Memory and Language.* Hove: Erlbaum.

[B33] Gombos V. A. (2006). The cognition of deception: the role of executive processes in producing lies. *Genet. Soc. Gen. Psychol. Monogr.* 132 197–214. 10.3200/MONO.132.3.197-214 17969998

[B34] Graesser A. C. (1981). *Prose Comprehension Beyond the Word.* New York, NY: Springer.

[B35] Graesser A. C. Nakamura G. V. (1982). “The impact of a schema on comprehension and memory,” in *The Psychology of Learning and Motivation* ed. Bower G. H. (New York, NY: Academic Press) 59–109.

[B36] Greathouse S. M. Kovera M. B. (2009). Instruction bias and lineup presentation moderate the effects of administrator knowledge on eyewitness identification. *Law Hum. Behav.* 33 70–82. 10.1007/s10979-008-9136-x 18594956

[B37] Grice H. P. (1975). “Logic and conversation,” in *Syntax and Semantics* eds Cole P. Morgan J. L. (New York, NY: Academic Press) 41–58.

[B38] Hamm S. Bredenkamp J. (2004). “Working memory and slips of the tongue,” in *Multidisciplinary Approaches to Language Production* eds Pechmann T. Habel C. (New York, NY: Mouton) 573–600.

[B39] Hauch V. Blandón-Gitlin I. Masip J. Sporer S. L. (2015). Are computers effective lie detectors? A meta-analysis of linguistic cues to deception. *Pers. Soc. Psychol. Rev.* 19 307–342. 10.1177/1088868314556539 25387767

[B40] Herrmann T. (1982). *Sprechen und Sitation [Speaking and Situation].* New York, NY: Springer.

[B41] Horowitz M. W. Newman J. B. (1964). Spoken and written expression: an experimental analysis. *J. Abnormal Soc. Psychol.* 6 640–647. 10.1037/h0048589 14170048

[B42] Ibabe I. Sporer S. L. (2004). The influence of question form on the accuracy and confidence of memories for central and peripheral action and descriptive details of an event. *Appl. Cogn. Psychol.* 18 711–726. 10.1002/acp.1025

[B43] Kellogg R. T. (2007). Are written and spoken recall of text equivalent? *Am. J. Psychol.* 120 415–428. 17892086

[B44] Kleider H. M. Pezdek K. Goldinger S. D. Kirk A. (2008). Schema-driven source misattribution errors: remembering the expected from a witnessed event. *Appl. Cogn. Psychol.* 22 1–20. 10.1002/acp.1361

[B45] Köhnken G. (1990). *Glaubwürdigkeit [Credibility].* München: Psychologie Verlags Union.

[B46] Lampinen J. M. Meier C. Arnal J. A. Leding J. K. (2005). Compelling untruths: content borrowing and vivid false memories. *J. Exp. Psychol. Learn. Mem. Cogn.* 31 954–963. 10.1037/0278-7393.31.5.954 16248744

[B47] Lane J. D. Wegner D. M. (1995). The cognitive consequences of secrecy. *J. Pers. Soc. Psychol.* 69 237–253. 10.1037/0022-3514.69.2.237

[B48] Lane L. W. Liersch M. J. (2012). Can you keep a secret? Increasing speakers’ motivation to keep information confidential yields poorer outcomes. *Lang. Cogn. Process.* 27 462–473. 10.1080/01690965.2011.556348

[B49] Leins D. Fisher R. P. Ross S. J. (2013). Exploring liars’ strategies for creating deceptive reports. *Legal Criminol. Psychol.* 18 141–151. 10.1111/j.2044-8333.2011.02041.x

[B50] Levine T. R. (2014). Truth-default theory (TDT): a theory of human deception and deception detection. *J. Lang. Soc. Psychol.* 33 378–392. 10.1177/0261927X14535916

[B51] Levine T. R. McCornack S. A. (2014). Theorizing about deception. *J. Lang. Soc. Psychol.* 33 431–440. 10.1177/0261927x14536397

[B52] Lindsay D. S. (2008). “Source monitoring,” in *Cognitive Psychology of Memory: A Comprehensive Reference* ed. Roediger H. L. (Oxford: Elsevier) 325–348.

[B53] Loftus E. F. (2005). Planting misinformation in the human mind: a 30-year investigation of the malleability of memory. *Learn. Mem.* 12 361–366. 10.1101/lm.94705 16027179

[B54] Luria G. Rosenblum S. (2010). Comparing the handwriting behaviours of true and false writing with computerized handwriting measures. *Appl. Cogn. Psychol.* 24 1115–1128. 10.1002/acp.1621

[B55] Marian V. Neisser U. (2000). Language-dependent recall of autobiographical memories. *J. Exp. Psychol. Gen.* 129 361–368. 10.1037/0096-3445.129.3.361 11006905

[B56] Masip J. Herrero C. (2013). What would you say if you were guilty? ‘Suspects’ strategies during a hypothetical Behavior Analysis Interview concerning a serious crime. *Appl. Cogn. Psychol.* 27 60–70. 10.1002/acp.2872

[B57] Masip J. Sporer S. L. Garrido E. Herrero C. (2005). The detection of deception with the reality monitoring approach: a review of the empirical evidence. *Psychol. Crime Law* 11 99–122. 10.1080/10683160410001726356

[B58] McCornack S. A. Morrison K. Paik J. E. Wisner A. M. Zhu X. (2014). Information manipulation theory 2: a propositional theory of deceptive discourse production. *J. Lang. Soc. Psychol.* 33 348–377. 10.1177/0261927x14534656

[B59] Meservy T. O. Jensen M. L. Kruse J. Twitchell D. P. Tsechpenakis G. Burgoon J. K. (2005). Deception detection through automatic, unobtrusive analysis of nonverbal behavior. *IEEE Intell. Syst.* 20 36–43. 10.1109/MIS.2005.85

[B60] Miller G. A. Galanter E. Pribram K. H. (1960). *Plans and the Structure of Behavior.* New York, NY: Holt.

[B61] Moscovitch M. (1994). Cognitive resources and dual-task interference effects at retrieval in normal people: the role of the frontal lobes and medial temporal cortex. *Neuropsychology* 8 524–534. 10.1037/0894-4105.8.4.524

[B62] National Research Council (2003). *The Polygraph and Lie Detection.* Washington, DC: The National Academies Press.

[B63] Ormerod T. C. Dando C. J. (2014). Finding a needle in a haystack: toward a psychologically informed method for aviation security screening. *J. Exp. Psychol. Gen.* 144 76–84. 10.1037/xge0000030 25365531

[B64] Pennebaker J. W. Chew C. H. (1985). Behavioral inhibition and electrodermal activity during deception. *J. Pers. Soc. Psychol.* 49 1427–1433. 10.1037/0022-3514.49.5.1427 4078683

[B65] Read J. D. (1996). From a passing thought to a false memory in 2 minutes: confusing real and illusory events. *Psychon. Bull. Rev.* 3 105–111. 10.3758/BF03210749 24214811

[B66] Read J. D. Connolly D. A. (2007). “Effects of delay on memory for witnessed events,” in *Handbook of Eyewitness Psychology: Memory for Events* Vol. 1 eds Toglia M. P. Read J. D. Ross D. F. Lindsay R. C. L. (Mahwah, NJ: Erlbaum Associates) 117–155.

[B67] Reinhard M.-A. Sporer S. L. Scharmach M. Marksteiner T. (2011). Listening, not watching: situational familiarity and the ability to detect deception. *J. Pers. Soc. Psychol.* 101 467–484. 10.1037/a0023726 21707196

[B68] Reyna V. F. Brainerd C. J. (1995). Fuzzy-trace theory: an interim synthesis. *Learn. Individ. Diff.* 7 1–75. 10.1016/1041-6080(95)90031-4

[B69] Rojahn K. Pettigrew T. F. (1992). Memory for schema-relevant information: a meta-analytic resolution. *Br. J. Soc. Psychol.* 31 81–109. 10.1111/j.2044-8309.1992.tb00958.x 1535823

[B70] Rosenthal R. (2002). Covert communication in classrooms, clinics, courtrooms, and cubicles. *Am. Psychol.* 57 839–849. 10.1037/0003-066X.57.11.839 12564183

[B71] Sauerland M. Sporer S. L. (2011). Written vs. spoken eyewitness accounts: does modality of testing matter? *Behav. Sci. Law* 29 846–857. 10.1002/bsl.1013 22009462

[B72] Schank R. C. Abelson R. P. (1977). *Scripts, Plans, Goals, and Understanding: An Enquiry into Human Knowledge Structures.* Hillsdale, MI: Erlbaum.

[B73] Semin G. R. Fiedler K. (1991). The linguistic category model, its bases, applications and range. *Euro. Rev. Soc. Psychol.* 2 1–30. 10.1080/14792779143000006

[B74] Smith E. R. (1998). “Mental representation and memory,” in *The Handbook of Social Psychology* eds Gilbert D. T. Fiske S. T. Lindzey G. (Boston, MA: McGraw-Hill) 391–445.

[B75] Spitznagel A. (1998). *Geheimnis und Geheimhaltung [Secret and Keeping Secrets].* Göttingen: Hogrefe.

[B76] Sporer S. L. (1997). The less travelled road to truth: verbal cues in deception in accounts of fabricated and self-experienced events. *Appl. Cogn. Psychol.* 11 373–397. 10.1002/(SICI)1099-0720(199710)11:5<373::AID-ACP461>3.0.CO;2-0

[B77] Sporer S. L. (2004). “Reality monitoring and the detection of deception,” in *Deception Detection in Forensic Contexts* eds Granhag P.-A. Stromwall L. (Cambridge: Cambridge University Press) 64–102. 10.1017/CBO9780511490071

[B78] Sporer S. L. (2008). Lessons from the origins of eyewitness testimony research in Europe. *Appl. Cogn. Psychol.* 22 737–757. 10.1002/acp.1479

[B79] Sporer S. L. Küpper B. (1995). Realitätsüberwachung und die Beurteilung des Wahrheitsgehaltes von Erzählungen: Eine experimentelle Studie [Reality monitoring and the judgment of credibility of stories: An experimental investigation]. *Z. Sozialpsychol.* 26 173–193.

[B80] Sporer S. L. Petermann N. (2011). Paraverbal cues to deception as a function of interview type. *Paper Presented at the Annual Conference of the American Psychology-Law Society* Miami, FL.

[B81] Sporer S. L. Schwandt B. (2006). Paraverbal indicators of deception: a meta-analytic synthesis. *Appl. Cogn. Psychol.* 20 421–446. 10.1002/acp.1190

[B82] Sporer S. L. Schwandt B. (2007). Moderators of nonverbal indicators of deception. *Psychol. Public Policy Law* 13 1–34. 10.1037/1076-8971.13.1.1

[B83] Sporer S. L. Sharman S. J. (2006). Should I believe this? Reality monitoring of accounts of self-experienced and invented recent and distant autobiographical events. *Appl. Cogn. Psychol.* 20 985–1001. 10.1002/acp1234

[B84] Sporer S. L. Walther A. (2006). Detecting deception with content cues: general vs. specific questions. *Paper Presented at the Meeting of the American Psychology-Law Society* Petersburg, FL.

[B85] Sporer S. L. Weinhäupl U. Niederstadt E. Krebs R. Wilhelmi J. (2003). Die Beurteilung der Glaubhaftigkeit erfundener und selbst erlebter Aussagen anhand des Linguistischen Kategorienmodells [Assessing the credibility of invented and self-experienced accounts on the basis of the linguistic category models]. *Paper Presented at the Fachgruppentagung Sozialpsychologie der Deutschen Gesellschaft für Psychologie* Heidelberg.

[B86] Stangor C. McMillan D. (1992). Memory for expectancy-congruent and expectancy-incongruent information: a review of the social and social developmental literatures. *Psychol. Bull.* 111 42–61. 10.1037/0033-2909.111.1.42

[B87] Steller M. Koehnken G. (1989). “Criteria-based statement analysis,” in *Psychological Methods for Investigation and Evidence* ed. Raskin D. C. (New York, NY: Springer-Verlag) 217–245.

[B88] Street C. H. (2015). ALIED: humans as adaptive lie detectors. *J. Appl. Res. Mem. Cogn.* 4 335–343. 10.1016/j.jarmac.2015.06.002

[B89] Thompson C. P. Skowronski J. S. Larsen S. F. Betz A. L. (1996). *Autobiographical Memory: Remembering What and Remembering When.* New York, NY: Lawrence Erlbaum Associates.

[B90] Tuckey M. R. Brewer N. (2003a). How schemas affect eyewitness memory over repeated retrieval attempts. *Appl. Cogn. Psychol.* 17 785–800. 10.1002/acp.906

[B91] Tuckey M. R. Brewer N. (2003b). The influence of schemas, stimulus ambiguity, and interview schedule on eyewitness memory over time. *J. Exp. Psychol. Appl.* 9 101–118. 10.1037/1076-898X.9.2.101 12877270

[B92] Undeutsch U. (1967). *Beurteilung der Glaubhaftigkeit von Aussagen [Assessing the Credibility of Testimonies] Handbuch der Psychologie: Forensische Psychologie.* Göttingen: Hogrefe 26–181.

[B93] Verschuere B. De Houwer J. (2011). “Detecting concealed information in less than a second: response latency-based measures,” in *Memory Detection* eds Verschuere B. Ben-Shakhar G. Meijer E. (New York, NY: Cambridge University Press) 46–62.

[B94] Verschuere B. Prati V. De Houwer J. (2009). Cheating the lie detector. *Psychol. Sci.* 20 410–413. 10.1111/j.1467-9280.2009.02308.x 19298261

[B95] von Restorff H. (1933). Über die wirkung von bereichsbildungen im spurenfeld [The effects of field formation in the trace field]. *Psychol. Forsch.* 18 299–334. 10.1007/BF02409636

[B96] Vredeveldt A. Hitch G. J. Baddeley A. D. (2011). Eye-closure helps memory by reducing cognitive load and enhancing visualization. *Mem. Cogn.* 39 1253–1263. 10.3758/s13421-011-0098-8 21491166

[B97] Vrij A. Granhag P. A. Porter S. B. (2010). Pitfalls and opportunities in nonverbal and verbal lie detection. *Psychol. Sci. Public Interest* 11 89–121. 10.1177/1529100610390861 26168416

[B98] Vrij A. Mann S. A. Fisher R. P. Leal S. Milne R. Bull R. (2008). Increasing cognitive load to facilitate lie detection: the benefit of recalling an event in reverse order. *Law Hum. Behav.* 32 253–265. 10.1007/s10979-007-9103-y 17694424

[B99] Walczyk J. J. (2014). A commentary on information manipulation theory 2: its place in deception research and suggestions for elaboration. *J. Lang. Soc. Psychol.* 33 424–430. 10.1177/0261927X14535395

[B100] Walczyk J. J. Harris L. L. Duck T. K. Mulay D. (2014). A social-cognitive framework for understanding serious lies: activation-decision-construction-action theory. *New Ideas Psychol.* 34 22–36. 10.1016/j.newideapsych.2014.03.001

[B101] Walczyk J. J. Igou F. P. Dixon A. P. Tcholakian T. (2013). Advancing lie detection by inducing cognitive load on liars: a review of relevant theories and techniques guided by lessons from polygraph- based approaches [Advance online publication]. *Front. Psychol.* 4:14 10.3389/fpsyg.2013.00014 PMC3561742 23378840

[B102] Walczyk J. J. Mahoney K. T. Doverspike D. Griffith-Ross D. A. (2009). Cognititve lie detection: response time and consistency of answers as cues to deception. *J. Bus. Psychol.* 24 33–49. 10.1007/s10869-009-9090-8

[B103] Walczyk J. J. Roper K. S. Seemann E. Humphrey A. M. (2003). Cognitive mechanisms underlying lying to questions: response time as a cue to deception. *Appl. Cogn. Psychol.* 17 755–774. 10.1002/acp.914

[B104] Walczyk J. J. Schwartz J. P. Clifton R. Adams B. Wei M. Zha P. (2005). Lying person-to-person about life events: a cognitive framework for lie detection. *Personnel Psychol.* 58 141–170. 10.1111/j.1744-6570.2005.00484.x

[B105] Wardlow Lane L. Groisman M. Ferreira V. S. (2006). Don’t talk about pink elephants! Speakers’ control over leaking private information during language production. *Psychol. Sci.* 17 273–277. 10.1111/j.1467-9280.2006.01697.x 16623681 PMC1868699

[B106] Wegner D. M. (1994). Ironic processes of mental control. *Psychol. Rev.* 101 34–52. 10.1037/0033-295X.101.1.34 8121959

[B107] Wegner D. M. (2009). How to think, say, or do precisely the worst thing for any occasion. *Science* 325 48–50. 10.1126/science.1167346 19574380

[B108] Wegner D. M. Ansfield M. Pilloff D. (1998). The putt and the pendulum: ironic effects of the mental control of action. *Psychol. Sci.* 9 196–199. 10.1111/1467-9280.00037

[B109] Wegner D. M. Lane J. D. Dimitri S. (1994). The allure of secret relationships. *J. Pers. Soc. Psychol.* 66 287–300. 10.1037/0022-3514.66.2.287

[B110] Wellman H. M. Cross D. Watson J. (2001). Meta-analysis of theory-of-mind development: the truth about false belief. *Child Dev.* 72 655–684. 10.1111/1467-8624.00304 11405571

[B111] Wicklund R. A. (1975). “Objective self-awareness,” in *Advances in Experimental Social Psychology* Vol. 8 ed. Berkowitz L. (New York, NY: Academic Press) 233–275.

[B112] Wimmer H. Perner J. (1983). Beliefs about beliefs: representation and constraining function of wrong beliefs in young children’s understanding of deception. *Cognition* 13 103–128. 10.1016/0010-0277(83)90004-5 6681741

[B113] Zuckerman M. DePaulo B. M. Rosenthal R. (1981). “Verbal and nonverbal communication of deception,” in *Advances in Experimental Social Psychology* ed. Berkowitz L. (New York, NY: Academic Press) 1–57.

